# Nurses’ experiences of presenteeist behaviors in the hospital

**DOI:** 10.1590/0034-7167-2024-0331

**Published:** 2025-08-08

**Authors:** Lenícia Cruz Soares, Adrize Rutz Porto

**Affiliations:** I Universidade Federal de Pelotas. Pelotas, Rio Grande do Sul, Brazil

**Keywords:** Nurses, Presenteeism, Nursing Staff Hospital, Nursing, Qualitative Research., Enfermeros, Presentismo, Personal de Enfermería en Hospital, Enfermería, Investigación Cualitativa.

## Abstract

**Objectives:**

to understand nurses’ experiences regarding presenteeist behaviors in the hospital.

**Methods:**

a single holistic descriptive case study with a qualitative approach, with 32 nurses. Data were collected in three stages, through vignettes and in-depth individual interviews, audio-recorded and submitted to thematic analysis. The theoretical framework adopted was Christophe Dejours’ Psychodynamics of Work.

**Results:**

three topics emerged, revealing that the concept of presenteeism was not known among participants, but they recognized themselves experiencing presenteeist behaviors due to physical or psychological problems that, in some way, affected quality of work. The main reasons for presenteeist behavior were professional commitment and financial issues.

**Final Considerations:**

presenteeism manifested itself through physical distress, such as musculoskeletal pain, and psychological distress, such as burnout, arising from hospital work organization, seen as exhausting, marked by excessive demands, which can affect task performance, worker health and the quality of care provided.

## RESUMEN

**Objetivos:**

comprender las experiencias de enfermeros sobre conductas de presentismo en el hospital.

**Métodos:**

estudio descriptivo holístico de caso único, con enfoque cualitativo, con 32 enfermeros. Los datos fueron recolectados en tres etapas, a través de viñetas y entrevistas individuales en profundidad, grabadas en audio y sometidas a análisis temático. Se adoptó como marco teórico la Psicodinámica del Trabajo de Christophe Dejours.

**Resultados:**

emergieron tres temas, revelando que el concepto de presentismo no era conocido entre los participantes, pero reconocían experimentar conductas de presentismo, debido a problemas físicos o psicológicos que, de alguna manera, afectaban la calidad del trabajo. Las principales razones del comportamiento presentista fueron el compromiso profesional y las cuestiones financieras.

**Consideraciones Finales:**

el presentismo se manifiesta por sufrimiento físico, como dolor musculoesquelético, y sufrimiento psicológico, como fatiga, resultante de la organización del trabajo hospitalario, visto como agotador, marcado por exigencias excesivas, que pueden afectar el desempeño de las tareas, la salud del trabajador y la calidad de la atención brindada.

## INTRODUCTION

Presenteeism is a behavior in which professionals are physically present at work, but are unable to fully perform their work activities, due to their own physical or psychological problems. This behavior leads to a decrease in productivity and can have a major effect on work, even greater than absenteeism, when workers are absent from the workplace^(1)^. Presenteeism in nursing comes from the fact that the profession is particularly associated with care, a culture of teamwork, loyalty to co-workers and also professional identity, which gives nurses a sense of obligation to be present at work^(2)^.

However, nursing and healthcare professionals who work in hospital environments face daily situations of stress, pressure, work overload, exhaustion, lack of materials and equipment^(3)^, emotional exhaustion, frustration, dissatisfaction with the results of their work, lack of recognition, and conflicts^(4)^. In this context, health workers are susceptible to physical and mental illness, and the incidence of occupational stress is predominant among professionals with higher education^(3)^.

Mental health at work is therefore associated with the way professionals perceive aspects related to their work activity^(5)^. At the same time, presenteeism is a multidimensional phenomenon that is highly prevalent among healthcare workers^(6,7)^, and its difficult detection may be justified by the lack of information about this occupational disease, which leads to impaired performance, leading to possible distractions and serious errors in professional practice^(7)^.

Therefore, presenteeism may have intersections with distress at work, studied by Christophe Dejours, a French psychiatrist and psychoanalyst, whose research addresses topics in psychopathology, with his object of study being the psychic life at work. His studies and professional experience led him to the construction of a theoretical-methodological model called “Psychodynamics of Work”, which encompasses distress at work and mental pathologies related to it, as well as conditions in which work is a source of pleasure, and can play a role in health construction^(8)^.

Thus, the relevance of this study lies in the fact that the main causes of presenteeism refer to factors that compromise healthcare professionals’ mental health, especially nursing professionals, in hospital environments, such as stress, emotional exhaustion or depression^(9-13)^. Furthermore, presenteeism can aggravate existing health problems and increase the risk of subsequent illness and absenteeism as well as impair work capacity^(14-16)^ and cause serious harm to professionals’ physical, emotional and work lives^(15)^.

Although scientific production on the subject has been increasing in recent years, studies investigating presenteeism in healthcare professionals are still few and far between, especially in Brazil^(6,17,18)^. However, there are several factors that influence the increase or decrease in presenteeism, which can be managed by the occupational healthcare service, in order to mitigate the consequences of the existence of this phenomenon^(6)^. Thus, the guiding question of this study was: what are nurses’ experiences regarding presenteeist behaviors in hospital work?

## OBJECTIVES

To understand nurses’ experiences regarding presenteeist behaviors in the hospital.

## METHODS

### Ethical aspects

The research was approved by the Research Ethics Committee of the *Universidade Federal de Pelotas* (UFPel) School of Nursing, and complied with the ethical aspects established by Resolution 466/2012 of the Brazilian National Health Council. The Informed Consent Form was obtained from all individuals involved in the study in writing. They were identified by Arabic numerals, followed by the letters “V” for the Vignette data collection technique, or “I” for the Interview data collection technique. Since there are two situations in the vignettes, they were represented as VI and VII, the same occurring with the interviews, II and III. Thus, the codes are 1VI, 1VII, 1II, 2VI, 4VII, 7III, etc.

### Theoretical-methodological framework

The Theory of Psychodynamics of Work was adopted as the theoretical framework for this research, as it is a suitable approach for understanding the dynamics of work situations and possible harm to workers’ health^(8)^.

### Study design

This is a single holistic descriptive case study with a qualitative approach^(19)^. In order to qualify the study production writing, the COnsolidated criteria for REporting Qualitative research (COREQ) guidelines were adopted^(20)^.

### Study setting

This study was developed at the UFPel Teaching Hospital (TH), which has been managed by the *Empresa Brasileira de Serviços Hospitalares* (EBSERH) since 2014. Most TH workers are linked to the EBSERH network; however, there are still employees of the Single Legal Regime (RJU) working at the hospital.

### Data source

Nurses assigned to the inpatient wards that make up the Nursing Management Support Unit (In Portuguese, *Unidade de Apoio a Gestão de Enfermagem* - UAGENF) of UFPel/TH, such as the medical clinic, emergency and urgency network II and emergency and urgency network III, were invited to participate in the study. The selection criterion was to work at UFPel/TH, as a nurse, assigned to UAGENF for at least six months. Thus, 33 nurses were approached, and all agreed to participate in the study. However, there was one withdrawal after completing stage I of data collection (vignettes), leaving 32 participants.

### Data collection and organization

The vignettes and interviews were subjected to a sensitization study^(21)^ with nurses from another unit, in order to ascertain their suitability before being applied to the participants of this study. Data collection was carried out at nurses’ own work location and time, in a private room in the unit, where privacy and favorable conditions were provided so that they could answer the questionnaires. It took place in April and May 2023, conducted by the main author, with experience in qualitative research, and followed three stages:

Stage I - Vignettes: participants were given an instrument containing two vignettes (plots) and questions to identify their perceptions about such plots, which were answered in writing by participants and returned on the same day, or later, depending on workers’ availability. A vignette consists of a brief description of a situation, real or fictitious, structured in such a way as to attract attention, produce sensations and obtain information about respondents’ perceptions, attitudes or knowledge about a given phenomenon^(22)^. The vignettes were prepared by the authors themselves, based on their professional experiences.

Stage II - Interview I: an in-depth individual interview was conducted to identify socio-professional characteristics and aspects related to the work context^(23)^. This occurred after the instrument completed in stage I was returned. It was audio-recorded and lasted an average of 45 minutes.

Stage III - Interview II: a second individual interview was scheduled in order to deepen the discussion on the research topic, since in interview I there was not enough significant content regarding presenteeism. It took place a few days (three to seven days) after participants had had contact with the subject, providing time for the mobilization of feelings, doubts and reflections that could then be addressed in greater depth^(24)^. The interview, at this stage, was also audio-recorded and lasted an average of 25 minutes.

### Data analysis

After transcribing the vignettes and interviews in full, the collected data were treated according to the thematic analysis proposal, consisting of six phases: familiarizing with the data; generating initial codes; searching for topics; reviewing topics; defining and naming the topics; and producing the report^(25)^. Thus, three topics resulted: (Re)cognizing presenteeism and distress at work by nurses; Nurses’ experiences of physical and psychological distress and consequent presenteeist behavior; Motivations for nurses’ presenteeist behavior.

## RESULTS

Of the 32 nurses who participated in all stages of data collection, 28 were female. Regarding their employment status, 29 nurses were employed by EBSERH; three nurses were employed by RJU, with an average of 30 years of work at the institution, while 15 participants had worked at the hospital for less than five years and the other 14 had between five and ten years of work at the study site; 27 nurses had only one employment contract, while five had two contracts; their ages ranged from 26 to 56 years (average of 39 years). Concerning skin color, 24 declared themselves to be white, one was black, and the others were brown. Regarding marital status, ten were in a stable union; ten were married; ten were single; two were divorced; 18 participants had no children; 11 had only one child; two had two children; and one had three children.

In relation to their own health, ten nurses denied any illness, but hypertension was reported by five nurses; hypothyroidism was reported by four nurses; obesity was also mentioned by four nurses; and depression, diabetes, asthma, arrhythmia, gastritis, migraine, osteoarthritis, varicose veins, cervical hernia, bronchiectasis, pituitary microadenoma, saphenous vein insufficiency, retinal detachment, facet syndrome and stroke sequels were mentioned once. Half of nurses reported some limitation in the performance of their work activities, such as pain, loss of strength, limited movement, difficulty walking, fatigue, edema and discomfort.

### Topic 1: (Re)cognizing presenteeism and distress at work by nurses

Most of study participants were unfamiliar with the concept of presenteeism. Only two nurses came close to the definition:

*Presenteeism is you being physically present alone at work!?* (18II)

*About presenteeism* [...] *if you feel sick, how are you working!? If you have some emotional or physical issue, but you are still working.* (25II)

After the concept of presenteeism at work, adopted in this research, was presented, most nurses recognized that they were experiencing presenteeism:

*It is something that is very present in our area* [...]*. When we name what we experience, it becomes much more elucidated. Having been introduced to this term clarifies a lot and a lot of things make sense afterwards.* (1III)

*I ended up coming to work, but it was as if I wasn’t here* [...]*. I found myself just coming, going to this hospital, doing my shift and leaving. Really, not getting involved in anything else.* (7II)

*It’s something that has always been present in my professional life, I’ve always experienced it, but I had no idea, not even of this term, presenteeism. We do so much in our daily lives, we present this so much. We experience presenteeism every day.* (11III)

It is clear that this behavior is frequently present in nurses’ work, who report distress when coming to work, in addition to admitting harm to their professional practice:

*Many times, I came to work, but, in fact, only physically, trying to do my best, trying not to leave patients unattended, but, many times, I was only physically present* [...]*. I came trying to make things better, thinking that everything was fine, but it wasn’t.* (3II)

*I’m experiencing this presenteeism at this moment in my life. It’s bad, how much it affects performance, it affects professional life.* (3III)

*I came because I knew there was no one to take my place* [...]*. It has happened several times that I was here because I had to be, physically. I came, but I was not focused on the work.* (22II)

*I stopped to realize how many people in my department suffer from this and it goes unnoticed. I think that sometimes, in the rush of everyday life, we don’t listen to our co-workers and sometimes we fail to realize how sick they are, how much they need help* [...]*. And all of this already causes a lot of problems for the team, for the work environment.* (28III)

Nurses are responsible for a variety of tasks, ranging from management functions as nursing team leaders to procedures and care as well as intervening and mediating with the multidisciplinary health team. Thus, depending on the conditions of the work organization in the place where they work, nurses may experience stressful and exhausting situations, which precede presenteeism:

*Many times, I am physically present and I am doing things mechanically, as I am used to doing. Especially now, I am in a phase of great emotional and physical exhaustion* [...]*. This describes presenteeism perfectly.* (12II)

*When we are not feeling well, we do not perform tasks as well as we would like. I have frequent back pain, because we are always helping to move patients, taking patients for exams* [...]*. Many times, I come because I do not like to write medical certificates. We take medicine and go to work.* (30II)

Likewise, when a professional is dissatisfied with the place where they work, this also contributes to presenteeist behavior, as stated by one of the nurses:

*From the moment you are in a place where you are not satisfied, you don’t feel that you are 100 percent professionally there, I think the word is literally presenteeism. And that is what I feel a lot here where I am.* (16III)

Among the reports collected, it was found that nurses’ presenteeism is often not so visible, especially during and after the COVID-19 pandemic, as it was a period of uncertainty, distress and physical and mental overload:

*I think it became more apparent after the pandemic. If you talk to your co-workers, you see that you’re not the only one in this boat* [...]*, it’s a chronic thing, a chronic fatigue, because it never goes away* [...]*. This is so common, this is our routine. Sometimes, we don’t even realize it, we don’t even stop to think about how neglectful we are with ourselves.* (11II)

### Topic 2: Nurses’ experiences of physical and psychological distress and consequent presenteeist behavior

Study participants reported fatigue, stress, depression and emotional overload associated with working conditions, imposed rhythms, demands, interpersonal relationships, etc. The way work is organized in the hospital institution, therefore, is directly related to the distress experienced by nurses, which, in turn, will be a determinant of presenteeist behavior:

*I’ve come to work several times not feeling well* [...]*. I wasn’t feeling well mentally.* (16II)

*Due to these musculoskeletal problems that I have* [...]*, I came because the period of the certificate had already passed and I was still in pain. This happened several times.* (18II)

*I believe I had burnout, but I didn’t have a diagnosis. All the symptoms characterized it because I would get very anxious before work, I didn’t feel good about going to work. Whenever I thought, “I have to go to work”, I would feel bad. I was completely discouraged about working.* (20II)

*I’ve gone to work sick before, because I don’t like to miss work for nothing. Now, when it’s emotional, it’s more complicated, because you’re there, but your head is racing.* (23II)

*I came to work mentally shaken* [...]*. There is no such thing as “I came to work and forgot my whole life outside”. No, your problems follow you. I came to work, but I wasn’t one hundred percent.* (28II)

*I’ve come to work in pain several times, and I’ve taken medication for pain. Especially when I worked during the day, because there were no employees to hire and the demand was huge. It was difficult, I came in pain anyway.* (29II)

It is worth noting that, in some statements, absenteeism was identified as a consequence of presenteeism. In these cases, workers have an illness or health problem (whether mental or physical) that can be aggravated if there is no adequate treatment, resulting in absence from work:

*There is a much more psychological issue, of escaping from that situation, than actually being physically ill. I see from the absences and sick notes,* [...] *we see in absenteeism the issue of presenteeism, because, in presenteeism, I am there with my body, but I am not there with my head. And the sick note is a form of escape, at least I justify not being able to be there physically, because my head has not been there for hours.* (10III)

*He will take time off because he will start to feel pain, he will start to write a sick note [...], he will have to take time off to take care of himself because if he continues, he will continue to injure himself more and more.* (26VI)

This situation, in addition to having a negative impact on professionals’ quality of life, can lead to failures in the execution of tasks, reduced productivity and compromised quality of care provided to patients:

*It is a common problem that we do not realize, but that happens every day around us. We often notice that some professionals are distracted at work and this harms not only their work, but the work of the group as a whole.* (9III)

*It* [presenteeism] *is much more present in my daily life than I imagined* [...]*. We don’t even carry out the tasks we have to carry out, because we are not fully present in the environment due to tiredness, fatigue, stress, external motivations that also hinder us.* (12III)

*I see that the hospital is working with teams that are sick, with several health problems, with many sick leave certificates* [...]*, overloading the people who are sick. So, if they already have a health problem, they end up making the problem worse, then not coming to work and generating other sick leave certificates.* (30II)

Another point observed in this study is the fact that absence from work seems to be justified only in the case of an adverse, aggravated health condition of a more organic/physical dimension:

*I can count the number of sick notes on my fingers. I only won’t go if I’m really sick.* (10II)

*When I saw that it was no longer possible, I had to leave one day in the middle of a shift, because I had a very strong headache.* (11II)

*Our co-worker just returned from sick leave. This time, she stayed home because she was literally physically unable to come* [...]*. She stopped coming because she was very ill.* (16III)

Thus, when distress presents itself in a more subjective way, related to psychological issues, for example, it did not seem to be valid/recognized as a reason for being absent from work:

*Many times, I came to work, but in reality* [...] *I was only physically present, with my mental health very compromised. We are not always well, but I don’t see any problem in being here, in doing what I do.* (3II)

*For mental health reasons, I didn’t let myself take time off work. I came even though I knew something wasn’t right.* (24II)

### Topic 3: Motivations for nurses’ presenteeist behavior

Presenteeist behavior can manifest itself for several reasons. The main ones, according to this research, are due to professionals’ “commitment” to their duty, or their sense of “responsibility”, and due to “financial issues”, in order to avoid salary deductions and reduction of family income:

*Sometimes I’m in a little pain, but I never show it* [...] *I have this sense of obligation.* (12II)

*Those who are truly committed will come and not miss work. They will only miss work if they see that things are really bad and that they won’t be able to work.* (21II)

*I couldn’t take time off because my salary would go down. If you don’t come to work, you don’t get night shift pay. If you go on leave, you often don’t get paid for a period of time.* (28II)

Other justifications frequently given by participants for such behavior are “avoiding overload” from co-workers, “fear of punishment and insecurity”, in addition to maintaining “patient care”.

*I’m not well, but if I don’t go, it will create an overload and those patients will not receive adequate treatment because the team will be overloaded.* (4II)

*I think people are afraid of being punished; they are sometimes even afraid of being moved to another department or even losing their work shift.* (15II)

*It has happened that I didn’t provide a medical certificate, precisely so as not to harm the team.* (30II)

Furthermore, the following situations were mentioned in which professionals adopt a presenteeist attitude: “not wanting to stay at home”; receiving “support from co-workers”; “difficulty in obtaining a medical certificate”; and “avoiding judgment”:

*It could be a commitment to work, an obligation, a financial need. Often, it could also be a lack of desire to stay at home.* (13II)

*Depending on the problem she has at home, it is better for her to come to work than to stay there with the problem.* (17II)

*This has happened to me a few times. I avoid demonstrating for a number of reasons, including what people say, to avoid gossip.* (18II)

*I really come because of the team, I really like my co-workers. I feel very welcomed by them* [...]*, they make me feel here even when I’m sick.* (19II)

*Sometimes, they even come because they can’t get a medical certificate. They even feel bad, but they can’t get a medical certificate for that day and they don’t want to be absent.* (21II)

## DISCUSSION

This study revealed that the concept of presenteeism was not yet known among participants. However, after being introduced to the term, nurses recognized that they had experienced situations in which they were present in work environments, unable to fully perform their work activities due to physical or psychological problems. They even admitted that this behavior somehow affected the quality of their work. The results of this research converge with the concept that presenteeism is considered an occupational and psychosocial phenomenon that affects professionals in their work environment, compromising their productivity, harming their health condition^(7)^ and also impacting negative results for patients, nurses and health organizations^(12)^.

The nurses in the study explained that there is a close relationship between work and the process of illness among professionals. Dejour’s theoretical framework allowed us to understand the experiences of distress reported, considering that the subjective relationship with work plays an important role in the processes involved, both in health construction and in workers’ psychiatric and psychosomatic decompensations^(8)^.

Specific working conditions were shown to be intertwined with nurses’ illness, in which, in the present study, hypertension was reported by five nurses, hypothyroidism and obesity, each by four nurses, as well as the lack of adequate staff, resulting in presenteeist behaviors. Regarding the organization of nursing work in hospital environments, it is clear that this involves exhausting and stressful activities, among which are intense work rhythms, working for long periods in the same position or in inadequate positions, performing the same task repeatedly, the high emotional burden resulting from direct contact with patients (and family members), the process of illness, death/dying, etc. Such activities require manual and psychosocial skills, such as the ability to maintain attention and concentration to avoid iatrogenic events, relationships with the multidisciplinary team and patients/family members, in addition to exposure of workers to occupational, ergonomic, biological, chemical and physical risks^(26)^. Another point highlighted in this research is that presenteeism was exacerbated during the most critical period of the COVID-19 pandemic, given that nursing profession characteristics require these professionals to spend more time with patients, placing them as the “front line” in the fight against COVID-19^(27,28)^.

Thus, working conditions, relationships between managers and workers, the degree of accessibility to dialogue with managers, staffing, among others, are crucial factors in the emergence of presenteeist behavior^(15)^. In view of this, presenteeism is considered a complex and multifactorial issue, resulting from both physical and psychological problems, with stress and emotional exhaustion being among the main causes of presenteeism, which compromise professionals’ health^(9-12)^. Participants in this investigation expressed experiencing fatigue, stress and emotional overload associated with working conditions, imposed rhythms, demands, among others.

Absenteeism was sometimes identified by participants as a consequence of presenteeism, attributing to it a role as a trigger for absence from work. Indeed, presenteeism can aggravate existing health problems and increase the risk of subsequent illness and absenteeism as well as impair work ability^(16)^.

It is also worth noting, in participants’ statements, the fact that absence from work seems to be justified only in the case of an aggravated health condition and of an organic/physical dimension, because when distress was associated with psychological issues, it was not valid/recognized as a reason to be absent from work. In the same direction, it is made clear in the theoretical framework that only physical distress is recognized by work organization, while mental distress (especially anxiety) is not accepted in the workplace^(29)^. Therefore, for an individual to achieve disability status, they must have a characterized mental illness, which leads to the medicalization process^(29)^.

There are several reasons why presenteeist behavior may manifest itself. The main ones, according to this research, are due to professionals’ commitment to their duty, or their sense of responsibility, and due to financial issues, in order to avoid salary deductions and reduction in family income. Other justifications are: avoiding overloading co-workers; fear of punishment from superiors and insecurity; maintaining the quality of patient care; not wanting to stay at home; receiving support from co-workers even when not feeling well; difficulty in obtaining medical certificate; and avoiding judgment from others.

Presenteeism may be related to musculoskeletal symptoms, type of employment relationship^(15,30)^, work shift and location, and health conditions^(15)^. Professionals may adopt presenteeist behavior due to commitment to the team and because they believe that they will contribute to patients’ improvement^(30)^; to appear more committed to work; to be better seen and have a greater chance of progressing; for fear of being fired; for encouragement from superiors; to meet demanding objectives; to show illness and justify absence later; because they have been absent before; or for fear of being more limited in the future^(31)^.

Such situations are very characteristic of the purpose of work organization, since this is an aspect of the subjective dimension of work that is fundamental to the Psychodynamics of Work for understanding workers’ health and illness processes and through which the division of tasks and people in work environments is established^(32,33)^. Work organization causes certain imbalances; one of them refers to the increase in work rhythms and the demand for better performance^(29)^, thus causing an important influence on individuals’ mental health, since, regardless of the organizational model adopted in the production process, this has implications for workers’ health. From the moment workers are unable to adjust the task according to their needs and desires, mental distress begins^(29)^. Under these circumstances, the desire for production overcomes the desire for man, giving rise to a feeling of displeasure and tension^(33)^.

This experience of distress and struggle against the factors that push workers towards mental illness is intrinsic to presenteeist behavior, and each worker can manifest it in different ways, depending on their level of commitment to the institution and team, sense of belonging, sense of responsibility, their own personal and professional education, and also according to other professionals’ behavior based on the history of construction of the profession and organization of nursing work, according to the statements of the nurses in this study. It is noteworthy, however, that the motivation for presenteeist behavior was the fact that they did not want to stay at home and receive support from co-workers when they were not feeling well, which encourages us to reflect on whether work environments, in these cases, are preferable to domestic ones so that going to work serves as an escape from stressful family situations and whether co-workers actually provide well-being and support.

In view of this, presenteeism constitutes a challenge, being considered a multifactorial, subjective and complex issue, in addition to a non-palpable condition that requires professionals to acknowledge its occurrence^(34)^, despite the pressures of nursing work organization, which expects professionals to attend work, except if they present physical manifestations, acute or aggravated health conditions. Thus, as noted in normalized statements, nurses attend work because they feel that their absence will overload their co-workers, who would blame them for work overload, in a relationship of interdependence of the team that demands the presence of workers, regardless of how much they are under distress.

Therefore, it is important to highlight the importance of raising awareness among workers about presenteeism, given that this is still a hidden occupational condition. Otherwise, a continuous cycle of illness and distress at work will continue, due to work organization itself, which does not always have enough people on leave to replace absent employees, which also points to the co-responsibility of healthcare institutions in the event of presenteeism.

Furthermore, due to the particularity of nursing work, presenteeism can adversely affect patients’ health safety, as it compromises quality of care, being associated with the possibility of errors, patient falls and a decrease in professionals’ attention in the development of work activities^(1,12,13,35)^.

### Study limitations

The research refers to the experiences of 32 nurses regarding their health and illness situations in hospital environments. Therefore, the results presented here do not seek generalizations or effective solutions, since work organization may differ according to the healthcare institution and individuals dynamically experience new situations in their workplaces.

### Contributions to nursing, health, or public policy

The data collected showed that the organization of nurses’ work influences these professionals’ illness process, who experience presenteeism in various forms. However, presenteeism is a phenomenon that is difficult to detect, possibly due to its multidimensional nature and the lack of information about this occupational condition. Moreover, the social, professional, and organizational perspective of the relevance of nursing in direct and continuous patient care reinforces, as expressed by nurses in this study, that the duty to patients are placed above workers’ distress, regardless of the safety and quality conditions under which this professional will provide care to patients and family members. In view of this, we sought to expand knowledge about nurses’ presenteeism and its manifestations, in addition to highlighting the need for administrative bodies of healthcare institutions to pay attention to its existence and implement measures that minimize or eliminate the occurrence of this phenomenon in nurses’ work.

## FINAL CONSIDERATIONS

After the concept of presenteeism was presented to nurses, they began to report experiences of this behavior, which manifested itself through physical distress, such as musculoskeletal pain, mainly, and psychological distress, such as fatigue, anxiety, overload, demotivation, dissatisfaction, arising from the inherent characteristics of nursing profession work dynamics and the way in which hospital work is organized, seen as exhausting, marked by intense work rhythms and excessive demands, which can affect task performance, lead to harm to workers’ health and a reduction in the quality of care provided.

## Data Availability

The research data are available within the article.
