# Supplementary material for: Nurses’ experiences of presenteeist behaviors in the hospital
Source: Rev Bras Enferm. 2025 Aug 8;78(4):e20240331. doi: 10.1590/0034-7167-2024-0331 (PMC12334135; doi:10.1590/0034-7167-2024-0331)
Supplement: Translation

# Translation: Vivências de enfermeiros sobre comportamentos presenteístas no hospital

**DOI:** 10.1590/0034-7167-2024-0331pt

**Authors:** Lenícia Cruz Soares, Adrize Rutz Porto

**Affiliations:** I Universidade Federal de Pelotas. Pelotas, Rio Grande do Sul, Brasil

**Keywords:** Enfermeiros, Presenteísmo, Recursos Humanos de Enfermagem Hospitalar, Enfermagem, Pesquisa Qualitativa

## Abstract

**Objetivos:**

conhecer as vivências de enfermeiros sobre comportamentos presenteístas no hospital.

**Métodos:**

estudo de caso único holístico descritivo, de abordagem qualitativa, com 32 enfermeiros. Os dados foram coletados em três etapas, por meio de vinhetas e entrevistas individuais em profundidade, audiogravadas e submetidas à análise temática. Adotou-se, por referencial teórico, a Psicodinâmica do Trabalho de Christophe Dejours.

**Resultados:**

três temas emergiram, revelando que o conceito de presenteísmo não era conhecido entre os participantes, porém eles se reconheceram vivenciando comportamentos presenteístas, devido a problemas físicos ou psicológicos que, de alguma forma, afetavam a qualidade do trabalho. As principais razões do comportamento presenteísta foram compromisso profissional e questões financeiras.

**Considerações Finais:**

o presenteísmo se manifestou por sofrimentos físicos, como dores osteomusculares, e psíquicos, como estafa, advindos da organização do trabalho hospitalar, visto como desgastante, marcado por excesso de demandas, podendo afetar desempenho das tarefas, saúde do trabalhador e qualidade dos cuidados prestados.

## INTRODUÇÃO

O presenteísmo é um comportamento no qual os profissionais estão fisicamente presentes no trabalho, porém sem conseguir desempenhar plenamente suas atividades laborais, devido aos seus próprios problemas físicos ou psicológicos. Esse comportamento leva à diminuição da produtividade, podendo exercer grande efeito sobre o trabalho, até maior que o absenteísmo, quando o trabalhador está ausente no ambiente laboral^(1)^. O presenteísmo na enfermagem provém do fato de que a profissão está particularmente associada ao cuidado, à cultura do trabalho em equipe, à lealdade aos colegas e, também, à identidade profissional, o que confere ao enfermeiro um senso de obrigação de se fazer presente no trabalho^(2)^.

Não obstante, os profissionais de enfermagem e de saúde que atuam no ambiente hospitalar convivem diariamente com situações de estresse, pressão, sobrecarga de trabalho, exaustão, falta de materiais e equipamentos^(3)^, desgaste emocional, frustração, descontentamento com o resultado do trabalho, falta de reconhecimento, e conflitos^(4)^. Nesse contexto, os trabalhadores de saúde são suscetíveis ao adoecimento físico e mental, sendo que a incidência de estresse ocupacional é preponderante entre os profissionais de nível superior^(3)^.

A saúde mental no trabalho está, portanto, associada à maneira como os profissionais percebem os aspectos relacionados à sua atividade laboral^(5)^. Paralelamente, o presenteísmo é um fenômeno de natureza multidimensional que apresenta alta prevalência nos trabalhadores de saúde^(6,7)^, e sua difícil detecção talvez seja justificada pela falta de informação sobre essa doença ocupacional, que conduz a um desempenho prejudicado, acarretando possíveis distrações e erros graves no exercício profissional^(7)^.

Sendo assim, o presenteísmo pode ter intersecções com o sofrimento no trabalho, estudado por Christophe Dejours, psiquiatra e psicanalista francês, cujas pesquisas abordam temas da área da psicopatologia, sendo seu objeto de estudo a vida psíquica no trabalho. Seus estudos e experiência profissional conduziram-no para a construção de um modelo teórico-metodológico denominado “Psicodinâmica do Trabalho”, que abrange sofrimento no trabalho e patologias mentais a ele relacionadas, bem como condições em que o trabalho é fonte de prazer, podendo desempenhar um papel na construção da saúde^(8)^.

Assim, a relevância deste estudo reside no fato de que as principais causas de presenteísmo se referem aos fatores que comprometem a saúde mental dos profissionais de saúde, em especial os profissionais de enfermagem, em ambientes hospitalares, tais como estresse, exaustão emocional ou depressão^(9-13)^. Além disso, o presenteísmo pode agravar os problemas de saúde existentes e aumentar o risco de doença e absenteísmo subsequentes, bem como prejudicar a capacidade de trabalho^(14-16)^ e ocasionar sérios danos à vida física, emocional e laboral dos profissionais^(15)^.

Embora a produção científica referente ao tema venha aumentando nos últimos anos, os estudos que investigam presenteísmo em profissionais de saúde ainda são poucos, principalmente no Brasil^(6,17,18)^. Todavia, diversos são os fatores influenciadores do aumento ou queda do presenteísmo, os quais podem ser manejados pelo serviço de saúde do trabalhador, a fim de atenuar as consequências da existência desse fenômeno^(6)^. Assim, a questão norteadora deste estudo foi: quais as vivências de enfermeiros sobre comportamentos presenteístas no trabalho hospitalar?

## OBJETIVOS

Conhecer as vivências de enfermeiros sobre comportamentos presenteístas no hospital.

## MÉTODOS

### Aspectos éticos

A pesquisa foi aprovada pelo Comitê de Ética em Pesquisa da Faculdade de Enfermagem da Universidade Federal de Pelotas (UFPel), e atendeu aos aspectos éticos estabelecidos pela Resolução n^o^ 466/2012 do Conselho Nacional de Saúde. O Termo de Consentimento Livre e Esclarecido foi obtido de todos os indivíduos envolvidos no estudo por escrito. Esses foram identificados por algarismos arábicos, seguido pelas letras “V”, para a técnica de coleta de dados da Vinheta, ou “E”, para a técnica de coleta de dados da Entrevista. Como há duas situações nas vinhetas, elas foram representadas como VI e VII, ocorrendo o mesmo com as entrevistas, EI e EII. Dessa maneira, os códigos são 1VI, 1VII, 1EI, 2VI, 4VII, 7EII, etc.

### Referencial teórico-metodológico

A Teoria da Psicodinâmica do Trabalho foi adotada como referencial teórico desta pesquisa, visto que é uma abordagem propícia para entender a dinâmica das situações de trabalho e os possíveis agravos à saúde do trabalhador^(8)^.

### Tipo de estudo

Trata-se de estudo de caso único holístico descritivo, de abordagem qualitativa^(19)^. A fim de qualificar a escrita da produção do estudo, foram adotadas as diretrizes do *COonsolidated criteria for REporting Qualitative research* (COREQ)^(20)^.

### Cenário do estudo

Este estudo foi desenvolvido no Hospital Escola (HE) da UFPel, sendo o hospital administrado pela Empresa Brasileira de Serviços Hospitalares (EBSERH) desde 2014. A maioria dos trabalhadores do HE é vinculada à rede EBSERH, no entanto ainda há servidores do Regime Jurídico Único (RJU) atuando no hospital.

### Fonte de dados

Foram convidados para participar do estudo os enfermeiros lotados nos setores de internação que compõem a Unidade de Apoio a Gestão de Enfermagem (UAGENF) do HE/UFPel, tais como clínica médica, rede de urgência e emergência II e rede de urgência e emergência III. O critério de seleção foi trabalhar no HE/UFPel, no cargo de enfermeiro, lotado na UAGENF há pelo menos seis meses. Dessa maneira, foram abordados 33 enfermeiros, sendo que todos aceitaram participar do estudo. No entanto, houve uma desistência após realizar a etapa I da coleta de dados (vinhetas), restando 32 participantes.

### Coleta e organização de dados

As vinhetas e as entrevistas foram submetidas a estudo de sensibilização^(21)^ com enfermeiros de outra unidade, a fim de averiguar sua adequabilidade antes de serem aplicadas aos participantes deste estudo. A coleta de dados foi realizada no próprio local e horário de trabalho do enfermeiro, em sala reservada na unidade, onde se proporcionaram privacidade e condições favoráveis para que este pudesse responder aos questionários. Ocorreu nos meses de abril e maio de 2023, conduzida pela autora principal, com experiência em pesquisa qualitativa, e seguiu três etapas:

Etapa I - Vinhetas: foi fornecido aos participantes um instrumento contendo duas vinhetas (enredos) e perguntas para identificar suas percepções sobre tais enredos, as quais foram respondidas por escrito pelo participante e devolvidas no mesmo dia, ou posteriormente, conforme disponibilidade do trabalhador. A vinheta consiste em uma descrição breve de uma situação, real ou fictícia, estruturada de modo a chamar atenção, produzir sensações e obter informações sobre percepções, atitudes ou conhecimentos dos respondentes sobre determinado fenômeno^(22)^. As vinhetas foram elaboradas pelas próprias autoras, a partir das suas experiências profissionais.

Etapa II - Entrevista I: foi realizada entrevista individual em profundidade para identificar características socioprofissionais e aspectos relacionados ao contexto laboral^(23)^. Essa ocorreu após a devolução do instrumento preenchido na etapa I. Foi audiogravada e teve duração média de 45 minutos.

Etapa III - Entrevista II: foi agendada uma segunda entrevista individual, a fim de aprofundar a discussão sobre o tema de pesquisa, pois, na entrevista I, não houve conteúdo suficiente e significativo em relação ao presenteísmo. Ocorreu alguns dias (três a sete dias) após o participante ter tido contato com o assunto, proporcionando tempo para a mobilização de sentimentos, dúvidas e reflexões que, então, poderiam ser abordadas com maior profundidade^(24)^. A entrevista, nesta etapa, também foi audiogravada e teve duração média de 25 minutos.

### Análise de dados

Após a transcrição das vinhetas e entrevistas na íntegra, os dados coletados foram tratados segundo a proposta de análise temática, composta por seis fases: familiarizar-se com os dados; gerar códigos iniciais; buscar temas; rever temas; definir e nomear os temas; e produzir o relatório^(25)^. Desse modo, resultaram três temas: (Re)conhecendo o presenteísmo e o sofrimento no trabalho pelos enfermeiros; Vivências de sofrimentos físicos e psicológicos dos enfermeiros e consequente comportamento presenteísta; Motivações para o comportamento presenteísta dos enfermeiros.

## RESULTADOS

Dos 32 enfermeiros que participaram de todas as etapas da coleta de dados, 28 deles são do sexo feminino. Em relação ao regime trabalhista, 29 enfermeiros são empregados celetistas da EBSERH; três enfermeiros são servidores do RJU, que detêm uma média de 30 anos de trabalho na instituição, ao passo que 15 participantes trabalham no hospital há menos de cinco anos e os outros 14 possuem entre cinco e dez anos de trabalho no local do estudo; 27 enfermeiros possuem apenas um vínculo empregatício, enquanto cinco possuem dois vínculos; a idade variou entre 26 e 56 anos (média de 39 anos). Quanto à cor da pele, 24 se autodeclararam de cor branca, uma, preta, e os demais, parda. Em relação ao estado civil, dez têm união estável; dez são casados; dez são solteiros; dois são divorciados; 18 participantes não têm filhos; 11 têm apenas um filho; dois têm dois filhos; e um tem três filhos.

Em relação à sua própria saúde, dez enfermeiros negam qualquer doença, mas hipertensão foi referida por cinco enfermeiros; hipotireoidismo foi referido por quatro enfermeiros; obesidade também foi mencionada por quatro enfermeiros; e depressão, diabetes, asma, arritmia, gastrite, enxaqueca, artrose, varizes, hérnia cervical, bronquiectasia, microadenoma hipofisário, insuficiência de safena, deslocamento de retina, síndrome facetária e sequela de acidente vascular cerebral foram citados uma vez. Metade dos enfermeiros relatou alguma limitação no desempenho de suas atividades laborais, tais como dor, perda de força, limitação de movimentos, dificuldade ao deambular, fadiga, edema e desconforto.

### Tema 1: (Re)conhecendo o presenteísmo e o sofrimento no trabalho pelos enfermeiros

Os participantes do estudo, em sua maioria, não conheciam o conceito de presenteísmo. Apenas dois enfermeiros se aproximaram da definição:

*Presenteísmo é tu estares de corpo presente só, no trabalho!?* (18EI)

*Sobre o presenteísmo* [...] *se tu te sentes doente, como é que tu estás trabalhando!? Se tu tens alguma questão emocional ou física, mas ainda assim estás trabalhando.* (25EI)

Após ter sido apresentado o conceito de presenteísmo no trabalho, adotado nesta pesquisa, a maioria dos enfermeiros se reconheceu vivenciando o presenteísmo:

*É algo que está muito presente na nossa área* [...]. *Quando a gente nomeia aquilo que a gente vive, fica muito mais elucidado. Ter sido apresentado a esse termo esclarece muito e muita coisa faz sentido depois.* (1EII)

*Eu acabava vindo trabalhar, mas era como se eu não estivesse aqui* [...]. *Eu me vi só na função de vir, entrar nesse hospital, cumprir meu horário e ir embora. Realmente, não me envolver em mais nada.* (7EI)

*É uma coisa que sempre esteve presente na minha vida profissional, sempre vivenciei, mas que eu não tinha noção, nem dessa nomenclatura, presenteísmo. Que a gente faz tanto no nosso dia a dia, apresenta tanto isso. A gente vivencia o presenteísmo todos os dias.* (11EII)

Percebe-se que esse comportamento é frequentemente presente no trabalho dos enfermeiros, os quais referem sofrimentos ao vir trabalhar, além de admitirem prejuízo no exercício profissional:

*Muitas vezes, eu vim trabalhar, mas, na verdade, só de corpo, tentando fazer o melhor sempre, tentando não deixar os pacientes desassistidos, mas, por muitas vezes, eu estava só de corpo presente* [...]. *Vim tentando amenizar, achando que estava tudo bem, mas não estava.* (3EI)

*Eu estou vivendo esse presenteísmo nesse momento da minha vida. É ruim isso, o quanto atrapalha no desempenho, atrapalha na vida profissional mesmo.* (3EII)

*Eu vinha, porque eu sabia que não tinha quem ficasse no meu lugar* [...]. *Já aconteceu várias vezes eu estar aqui porque tinha que estar, de corpo. Vinha, mas não ficava concentrada no serviço.* (22EI)

*Eu parei para perceber como tem pessoas com isso no meu setor e que passava imperceptível. Acho que, às vezes, a gente, na correria do dia a dia, passa sem escutar o colega e às vezes deixa de perceber o quanto ele está doente, o quanto ele está precisando de ajuda* [...]. (28EII)

Os enfermeiros são responsáveis por diversas atribuições, que vão desde as funções gerenciais, enquanto líder da equipe de enfermagem, até os procedimentos e cuidados assistenciais, além de intervir e intermediar junto à equipe multiprofissional de saúde. Assim, conforme as condições da organização do trabalho no local em que atua, o enfermeiro pode vivenciar situações desgastantes e estressantes, as quais antecedem o presenteísmo:

*Muitas vezes, eu estou de corpo presente e fazendo as coisas mecanicamente, como eu estou acostumada a fazer. Principalmente agora, eu estou numa fase com muita estafa emocional e física* [...]. *Me descreve perfeitamente o presenteísmo.* (12EI)

*Quando a gente não está bem, a gente não faz as tarefas tão bem como a gente gostaria. Eu tenho dores nas costas bem frequentes, em função da gente estar sempre auxiliando a mudar paciente, levar paciente para exames* [...]. *Muitas vezes, eu venho porque não gosto de ficar colocando atestado. A gente toma remédio e vai trabalhar.* (30EI)

Da mesma forma, quando o profissional está insatisfeito com o local em que trabalha, isto também contribui para o comportamento presenteísta, conforme declarou um dos enfermeiros:

*A partir do momento que você está num lugar que você não está satisfeito, não está sentindo que você está cem por cento profissionalmente ali, eu acho que literalmente a palavra é presenteísmo. E é isso que eu sinto muito aqui onde eu estou.* (16EII)

Constatou-se, entre os relatos coletados, o presenteísmo de enfermeiros que, muitas vezes não é tão visível, especialmente durante e após a pandemia de COVID-19, por ter sido um período de incertezas, sofrimento e sobrecarga física e mental:

*Eu acho que aflorou mais depois da pandemia. Se tu conversares com os colegas, tu vê que não é só tu que estás nesse barco* [...], *é uma coisa crônica, um cansaço crônico, porque nunca passa* [...]. *Isso é tão comum, isso é a nossa rotina. Às vezes, a gente nem se dá conta, nem para para pensar o quanto a gente é negligente com a gente mesmo.* (11EI)

### Tema 2: Vivências de sofrimentos físicos e psicológicos dos enfermeiros e consequente comportamento presenteísta

Os participantes do estudo relataram fadiga, estresse, depressão e sobrecarga emocional associados às condições de trabalho, aos ritmos impostos, cobranças, relações interpessoais, etc. A forma como se dá a organização de trabalho na instituição hospitalar, portanto, está diretamente relacionada ao sofrimento vivenciado pelos enfermeiros que, por sua vez, será determinante do comportamento presenteísta:

*Várias vezes já vim trabalhar não estando bem* [...]. *Não estava bem mentalmente.* (16EI)

*Em função desses problemas osteomusculares que eu tenho* [...], *eu vim porque já tinha passado o período do atestado e eu continuava com dor. Várias vezes aconteceu isso.* (18EI)

*Eu acredito que eu tive* Burnout*, mas eu não tive diagnóstico. Todos os sintomas caracterizavam isso porque eu ficava muito ansioso antes de trabalhar, eu não me sentia bem em ir trabalhar. Sempre quando eu pensava “Eu tenho que ir trabalhar”, eu já ficava mal. Eu tinha um desânimo total em trabalhar.* (20EI)

*Eu já fui trabalhar doente, porque eu não gosto de ficar faltando por bobagens. Agora, quando é o emocional, é mais complicado, porque tu está ali, mas a tua cabeça está a mil.* (23EI)

*Eu vinha trabalhar abalada mentalmente* [...]. *Não existe isso de “eu vim trabalhar e eu esqueci toda a minha vida lá fora”. Não, os teus problemas te acompanham. Eu vinha trabalhar, mas eu não estava cem por cento.* (28EI)

*Várias vezes eu já vim trabalhar com dor, já fiz medicação aqui para dor. Principalmente quando eu trabalhava de dia, porque não tinha funcionário para colocar e era aquela demanda imensa. Era difícil, eu vinha mesmo com dor.* (29EI)

Destaca-se que, em algumas falas, o absenteísmo foi identificado como uma consequência do presenteísmo. Nesses casos, o trabalhador apresenta uma doença ou problema de saúde (seja mental ou físico) que pode ser agravado se não houver um tratamento adequado, resultando na falta ao trabalho:

*Tem alguma questão muito mais psicológica, de fugir daquela situação, do que realmente estar doente fisicamente. Eu vejo pelos afastamentos e atestados,* [...] *a gente vê no absenteísmo a questão do presenteísmo, porque, no presenteísmo, eu estou ali com o corpo, mas não estou com a cabeça. E o atestado é uma forma de fuga, pelo menos eu justifico não poder estar lá fisicamente, porque minha cabeça não está mais há horas.* (10EII)

*Vai se afastar porque vai começar a sentir dores, vai colocar atestado* [...], *vai ter que se afastar para se cuidar porque se ele continuar vai cada vez se lesionando mais.* (26VI)

Essa situação, além de repercutir de maneira negativa sobre a qualidade de vida do profissional, pode levar à indução de falhas na execução das tarefas, redução na produtividade e comprometimento na qualidade da assistência prestada ao paciente:

*É um problema corriqueiro que não nos damos conta, mas que acontece todos os dias ao nosso redor. Muitas vezes, notamos que alguns profissionais estão dispersos no trabalho e isso prejudica não só o trabalho dele, mas o do coletivo.* (9EII)

*Ele* [o presenteísmo] *é muito mais presente no meu cotidiano do que eu imaginava* [...]. *A gente nem desenvolve as tarefas que a gente tem que desenvolver, porque não está totalmente presente ali no ambiente por cansaço, fadiga, estresse, motivações externas que atrapalham a gente também.* (12EII)

*Eu vejo que o hospital está trabalhando com equipes que estão doentes, com vários problemas de saúde, com muitos atestados* [...], *sobrecarregando as pessoas que estão. Então, se já estão com um problema de saúde, acabam piorando esse problema, depois não vindo trabalhar e gerando outros atestados.* (30EI)

Outro ponto observado neste estudo é o fato de que a ausência ao trabalho parece se justificar apenas em se tratando de uma condição de saúde adversa, agravada e de dimensão mais orgânica/física:

*Eu conto nos dedos os atestados. Eu só não vou se eu estiver doente realmente.* (10EI)

*Quando eu vi que não dava mais, eu tive que sair um dia na metade de um plantão, porque me deu uma dor muito forte na cabeça.* (11EI)

*A nossa colega acabou de retornar de um atestado. Dessa vez, ela ficou em casa porque não tinha condições físicas literalmente de vir* [...]. *Ela deixou de vir por estar muito mal.* (16EII)

Assim, quando o sofrimento se apresenta de maneira mais subjetiva, relacionado às questões psicológicas, por exemplo, parecia não ser válido/reconhecido como motivo para se ausentar do trabalho:

*Muitas vezes, eu vim trabalhar, mas, na verdade* [...] *estava só de corpo presente, com a saúde mental bem prejudicada. Nem sempre a gente está bem, mas eu não vejo problema em estar aqui, em fazer o que eu faço.* (3EI)

*Por saúde mental, eu não deixei me afastar do trabalho. Eu venho, mesmo sabendo que alguma coisa não está certa.* (24EI)

### Tema 3: Motivações para o comportamento presenteísta dos enfermeiros

O comportamento presenteísta pode se manifestar por diversas razões. As principais delas, segundo esta pesquisa, são devido ao “compromisso” do profissional com o seu dever, ou seu senso de “responsabilidade”, e devido às “questões financeiras”, a fim de evitar descontos no salário e redução da renda familiar:

*Às vezes, eu estou com um pouco de dor, mas nunca demonstro* [...] *eu tenho esse senso de obrigação.* (12EI)

*Quem é comprometido mesmo, vem e não vai faltar ao trabalho. Só vai faltar se ver que realmente está ruim e que não vai dar para trabalhar.* (21EI)

*Eu não podia me afastar, porque o salário diminui. Se tu não vens trabalhar, não ganha adicional noturno. Se tu vais entrar de licença, muitas vezes tu ficas um período sem receber.* (28EI)

Outras justificativas frequentemente apontadas pelos participantes para tal comportamento são “evitar a sobrecarga” dos colegas, “medo de punição e insegurança”, além de manter a “assistência ao paciente”.

*Eu não estou bem, mas se eu não for, vai gerar uma sobrecarga e aqueles pacientes não vão receber um tratamento adequado porque a equipe vai estar sobrecarregada.* (4EI)

*Eu acho que as pessoas têm medo de serem punidas; têm medo às vezes até de serem trocadas de setor ou até perder o turno de trabalho.* (15EI)

*Já aconteceu de eu não colocar atestado, justamente para não prejudicar a equipe.* (30EI)

Além disso, foram mencionadas as seguintes situações nas quais o profissional adota a atitude presenteísta: “não querer ficar em casa”; receber “apoio dos colegas”; “dificuldade em conseguir atestado” médico; e “evitar julgamentos”:

*Pode ser o compromisso que tenha com trabalho, a obrigação, a necessidade financeira. Muitas vezes, a não vontade de estar em casa também.* (13EI)

*Dependendo do problema que ela tem em casa, é melhor ela vir trabalhar do que ficar lá com o problema.* (17EI)

*Algumas vezes, já aconteceu isso. Eu evito demonstrar por ‘N’ motivos, até pelo que as pessoas falam, para evitar a falação.* (18EI)

*Eu venho mesmo por causa da equipe, eu gosto muito dos colegas. Eu me sinto bem acolhida com eles* [...], *me fazem estar aqui mesmo doente.* (19EI)

*Às vezes, até vem porque não tem como botar um atestado. Até se sente ruim, mas não tem como pegar um atestado daquele dia e não quer ficar com falta.* (21EI)

## DISCUSSÃO

Com este estudo, foi possível identificar que o conceito de presenteísmo ainda não era conhecido entre os participantes, no entanto, após terem sido apresentados ao termo, os enfermeiros se reconheceram vivenciando situações em que se fizeram presentes no ambiente de trabalho, sem conseguir desempenhar plenamente suas atividades laborais, devido aos problemas físicos ou psicológicos, admitindo, inclusive, que esse comportamento, de alguma forma, afetou a qualidade do trabalho. Os resultados da presente pesquisa convergem com a concepção de que o presenteísmo é considerado um fenômeno ocupacional e psicossocial que afeta o profissional no seu ambiente de trabalho, comprometendo sua produtividade, prejudicando sua condição de saúde^(7)^ e impactando também resultados negativos para pacientes, enfermeiros e organizações de saúde^(12)^.

Os enfermeiros do estudo expuseram que há uma estreita relação entre o trabalho e o processo de adoecimento dos profissionais. O referencial teórico dejouriano permitiu entender as vivências de sofrimento relatadas, tendo em vista que a relação subjetiva com o trabalho exerce um importante papel nos processos envolvidos, tanto na construção da saúde quanto nas descompensações psiquiátricas e psicossomáticas do trabalhador^(8)^.

As condições concretas para o trabalho se revelaram imbricadas no adoecimento do enfermeiro, em que, no presente estudo, foram relatados hipertensão, por cinco enfermeiros, hipotireoidismo e obesidade, cada uma por quatro enfermeiros, tal como a falta de quantitativo adequado de pessoal, repercutindo em comportamentos presenteístas. Tratando-se da organização do trabalho da enfermagem no ambiente hospitalar, é notório que este envolve atividades desgastantes e estressantes, entre as quais estão os ritmos laborais intensos, o fato de trabalhar por longos períodos na mesma posição ou em posições inadequadas, realizar repetidas vezes a mesma tarefa, a elevada carga emocional decorrente do contato direto com pacientes (e familiares), o processo de adoecimento, morte/morrer, etc. Tais atividades requerem habilidades manuais e psicossociais, como a capacidade de manter a atenção e a concentração para evitar iatrogenias, o relacionamento com equipe multiprofissional e paciente/familiares, além da exposição dos trabalhadores aos riscos ocupacionais, ergonômicos, biológicos, químicos e físicos^(26)^. Outro ponto ressaltado nesta pesquisa é que o presenteísmo se exacerbou no período mais crítico da pandemia de COVID-19, haja vista que as características da profissão de enfermagem requerem que esses profissionais permaneçam maior tempo ao lado dos pacientes, colocando-os como “linha de frente” no combate à COVID-19^(27,28)^.

Desse modo, as condições de trabalho, as relações entre gestores e trabalhadores, o grau de acessibilidade para dialogar com as chefias, o dimensionamento de pessoal, entre outros, são fatores cruciais no surgimento do comportamento presenteísta^(15)^. Em vista disso, considera-se o presenteísmo um tema complexo e multifatorial, decorrente tanto de problemas físicos quanto de problemas psicológicos, sendo que, entre as principais causas de presenteísmo, citam-se o estresse e a exaustão emocional, que comprometem a saúde dos profissionais^(9-12)^. Os participantes da presente investigação expressaram vivenciar fadiga, estresse e sobrecarga emocional associados às condições de trabalho, aos ritmos impostos, às cobranças, entre outros.

Algumas vezes, o absenteísmo foi identificado pelos participantes como consequência do presenteísmo, atribuindo a esse um papel de disparador da ausência no trabalho. De fato, o presenteísmo pode agravar os problemas de saúde existentes e aumentar o risco de doença e absenteísmo subsequentes, bem como prejudicar a capacidade de trabalho^(16)^.

Também merece atenção, na fala dos participantes, o fato de que a ausência no trabalho parece se justificar apenas em se tratando de uma condição de saúde agravada e de dimensão orgânica/física, pois quando o sofrimento estava associado às questões psicológicas, não era válido/reconhecido como motivo para se ausentar do trabalho. Nessa mesma direção, é explicitado no referencial teórico que apenas o sofrimento físico é reconhecido pela organização do trabalho, ao passo que o sofrimento mental (em especial, a ansiedade) não se admite no local de trabalho^(29)^. Sendo assim, para que o indivíduo consiga uma condição de invalidez, precisa apresentar uma doença mental caracterizada, o que leva ao processo de medicalização^(29)^.

Diversas são as razões pelas quais pode se manifestar o comportamento presenteísta. As principais delas, segundo esta pesquisa, são devido ao compromisso do profissional com o seu dever, ou seu senso de responsabilidade, e devido às questões financeiras, a fim de evitar descontos no salário e redução da renda familiar. Outras justificativas são: evitar a sobrecarga dos colegas; medo de punição de chefias e insegurança; manter a qualidade da assistência ao paciente; não querer ficar em casa; receber apoio dos colegas mesmo quando não está se sentindo bem; dificuldade em conseguir atestado médico; e evitar julgamentos alheios.

O presenteísmo pode estar relacionado aos sintomas osteomusculares, ao tipo de vínculo empregatício^(15,30)^, ao turno e local de trabalho, e às condições de saúde^(15)^. O profissional pode assumir o comportamento presenteísta ainda devido ao comprometimento com a equipe e por achar que vai contribuir para a melhora do paciente^(30)^; para parecer mais empenhado no trabalho; ficar melhor visto e com maior possibilidade de progredir; por receio de ser demitido; por incentivo das chefias; para cumprir objetivos exigentes; para se mostrar doente e justificar faltar depois; porque já faltou antes; ou por temer ficar mais limitado futuramente^(31)^.

Tais situações são bem características do propósito da organização de trabalho, uma vez que esta é um aspecto da dimensão subjetiva do trabalho fundamental à Psicodinâmica do Trabalho para a compreensão dos processos de saúde e adoecimento dos trabalhadores e pela qual se estabelece a divisão das tarefas e dos homens no ambiente laboral^(32,33)^. A organização do trabalho causa determinadas descompensações; uma delas se refere ao aumento dos ritmos de trabalho e à exigência de melhores desempenhos de rendimento^(29)^, acarretando, portanto, importante influência sobre a saúde mental dos indivíduos, visto que, independentemente do modelo organizacional adotado no processo produtivo, este tem implicações na saúde dos trabalhadores. A partir do momento que o trabalhador não consegue ajustar a tarefa de acordo com suas necessidades e desejos, tem-se início o sofrimento mental^(29)^. Sob essas circunstâncias, o desejo da produção supera o desejo do homem, originando um sentimento de desprazer e tensão^(33)^.

Essa vivência de sofrimento e luta contra os fatores que empurram o trabalhador para a doença mental está intrínseco ao comportamento presenteísta, e cada trabalhador pode manifestá-lo de diversas formas, segundo seu nível de comprometimento com a instituição e equipe, sentimento de pertença, seu senso de responsabilidade, sua própria educação pessoal e profissional, e, ainda, de acordo com o comportamento dos demais profissionais, a partir da história de construção da profissão e organização do trabalho da enfermagem, conforme os depoimentos dos enfermeiros do presente estudo. Destaca-se, contudo, que foi revelado pelos participantes como motivação para o comportamento presenteísta o fato de não querer ficar em casa e receber apoio dos colegas quando não estão se sentindo bem, o que instiga a refletir se o ambiente laboral, nesses casos, é preferível ao doméstico, de modo que ir trabalhar serve como fuga da situação estressora familiar e se os colegas de fato proporcionam bem-estar e suporte.

Em vista disso, o presenteísmo constitui-se em um desafio, sendo considerado um tema multifatorial, subjetivo e complexo, além de condição não palpável que requer o reconhecimento do profissional sobre sua ocorrência^(34)^, apesar das pressões da organização do trabalho da enfermagem, que espera que o profissional compareça ao trabalho, exceto se este apresentar manifestações físicas, condições de saúde agudas ou agravadas. Dessa maneira, conforme nota-se normalizado nas falas, o enfermeiro comparece ao trabalho, pois sente que sua ausência irá sobrecarregar o colega, o qual lhe atribuiria culpa pela sobrecarga de trabalho, em uma relação de interdependência da equipe que exige a presença do trabalhador, não importando como esteja sofrendo.

Por conseguinte, ressalta-se a importância da conscientização do trabalhador sobre o presenteísmo, diante de uma condição ocupacional oculta ainda. Caso contrário, mantém-se um ciclo contínuo de adoecimento e sofrimento no trabalho, em decorrência da própria organização do trabalho, que nem sempre dispõe de folguistas suficientes para substituição dos funcionários ausentes, o que aponta também para a corresponsabilidade das instituições de saúde diante da ocorrência do comportamento presenteísta.

Ademais, devido à particularidade do trabalho de enfermagem, o presenteísmo pode afetar adversamente a segurança da saúde dos pacientes, uma vez que compromete a qualidade da assistência, estando associado à possibilidade de erros, de quedas de pacientes e de diminuição da atenção do profissional no desenvolvimento das atividades laborais^(1,12,13,35)^.

### Limitações do estudo

A pesquisa refere-se à vivência dos 32 enfermeiros sobre as suas situações de saúde e adoecimento no ambiente hospitalar. Por isso, os resultados aqui apresentados não buscam generalizações ou soluções efetivas, visto que a organização do trabalho pode diferir de acordo com a instituição de saúde e que os indivíduos vivenciam dinamicamente novas situações em seus locais de trabalho.

### Contribuições para as áreas da enfermagem, saúde, ou políticas públicas

Os dados coletados mostraram que a organização do trabalho dos enfermeiros influencia o processo de adoecimento desses profissionais, os quais vivenciam o presenteísmo sob diversas formas. Contudo, o presenteísmo é um fenômeno de difícil detecção, possivelmente devido à sua natureza multidimensional e à falta de informação ainda sobre essa condição ocupacional. Além disso, a perspectiva social, profissional e organizacional da relevância da enfermagem no cuidado direto e contínuo ao paciente reforça, assim como manifestado pelos enfermeiros da presente investigação, que o dever com o enfermo é colocado acima do sofrimento do trabalhador, independentemente das condições de segurança e qualidade que esse profissional irá prestar os cuidados aos pacientes e familiares. Em vista disso, buscou-se ampliar o conhecimento acerca do presenteísmo de enfermeiros e suas manifestações, além de ressaltar a necessidade de que as instâncias administrativas das instituições de saúde atentem para a sua existência e implementem medidas que minimizem ou eliminem a ocorrência desse fenômeno no trabalho dos enfermeiros.

## CONSIDERAÇÕES FINAIS

Após o conceito de presenteísmo ter sido apresentado aos enfermeiros, iniciaram o relato de vivências desse comportamento, que se manifestou por sofrimentos físicos, como dores osteomusculares, principalmente, e psíquicos, como estafa, ansiedade, sobrecarga, desmotivação, insatisfação, advindos das características inerentes da dinâmica de trabalho da profissão da enfermagem e da forma como se dá a organização do trabalho hospitalar, visto como desgastante, marcado por ritmos laborais intensos e excesso de demandas, podendo afetar desempenho das tarefas, levar a prejuízos na saúde do próprio trabalhador e redução da qualidade dos cuidados prestados.

## Data Availability

Os dados de pesquisa estão disponíveis no corpo do artigo.
